# Concurrent activity of anammox and denitrifying bacteria in the Black Sea

**DOI:** 10.3389/fmicb.2012.00256

**Published:** 2012-07-19

**Authors:** John B. Kirkpatrick, Clara A. Fuchsman, Evgeniy Yakushev, James T. Staley, James W. Murray

**Affiliations:** ^1^School of Oceanography, University of Washington, SeattleWA, USA; ^2^Graduate School of Oceanography and Center for Dark Energy Biosphere Investigations, University of Rhode Island, NarragansettRI, USA; ^3^Norwegian Institute for Water ResearchOslo, Norway; ^4^Department of Microbiology, University of Washington, SeattleWA, USA

**Keywords:** anammox, denitrification, Black Sea, nirS, nitrogen, gene expression

## Abstract

After the discovery of ANaerobic AMMonium OXidation (anammox) in the environment, the role of heterotrophic denitrification as the main marine pathway for fixed N loss has been questioned. A 3 part, 15 month time series investigating nitrite reductase (*nirS*) mRNA transcripts at a single location in the Black Sea was conducted in order to better understand the activity of anammox and denitrifying bacteria. Here we show that both of these groups were active, as well as being concurrent in the lower suboxic zone over this time span. Their distributions, however, differed in that only expression of denitrification-type *nirS* was seen in the upper suboxic zone, where geochemistry was variable. Depth profiles covering the suboxic zone showed that the four groups of anammox-type sequences were expressed consistently in the lower suboxic zone, and were consistent with anammox 16 S rDNA gene profiles. By contrast, denitrifier-type *nirS* sequence groups were mixed; some groups exhibited consistent expression in the lower suboxic zone, while others appeared less consistent. Co-occurrence of both anammox and denitrifier expression was common and ongoing. Both types of transcripts were also found in samples with low concentrations of sulfide (>2 μM). Six major groups of denitrifier-type *nirS* transcripts were identified, and several groups of denitrifier-type *nirS* transcripts were closely related to sequences from the Baltic Sea. An increase in denitrifier-type *nirS* transcript diversity and depth range in October 2007 corresponded to a small increase in mixed layer net community productivity (NCP) as measured by O_2_/Ar gas ratios, as well as to an increase in N_2_ concentrations in the suboxic zone. Taken together, the variations in expression patterns between anammox and denitrification provide one possible explanation as to how near instantaneous rate measurements, such as isotope spike experiments, may regularly detect anammox activity but underreport denitrification.

## Introduction

Fixed nitrogen loss from marine systems balances N fixation, thereby exerting a long-term control over primary productivity and therefore climate (Altabet et al., 1995, 1999; Ganeshram et al., 1995), as well as anthropogenic influences. Loss of fixed N occurs via two microbial pathways: denitrification and the more recently discovered anammox process (ANaerobic AMMonium OXidation). While both require nitrite (NO_2_^−^), heterotrophic denitrification is reliant on organic C while anammox requires ammonium (NH_4_^+^). Since the discovery of environmental anammox in marine sediments (Thamdrup and Dalsgaard, 2002) and the water column (Dalsgaard et al., 2003; Kuypers et al., 2003), many groups have debated the relative role of these two processes in the environment. Anammox organisms have now been documented in many water column marine oxygen minimum zones (OMZs), and isotope labeling experiments have even shown in some cases a complete lack of denitrification (Schmid et al., 2007; Jensen et al., 2008; Lam et al., 2009). Other labeling studies have pointed towards a dominant contribution from denitrifiers (Ward et al., 2009), while DNA-based methods have suggested the potential for considerable variation in levels of heterotrophic denitrification (Jayakumar et al., 2009a). Conclusions regarding the dominance of one pathway over the other have thus been varied and conflicting (Lam et al., 2007; Schmid et al., 2007; Fuchsman et al., 2008; Lam et al., 2009; Ward et al., 2009; Bulow et al., 2010; Jensen et al., 2011). It has also been shown that it is possible to construct an N cycle where the role of heterotrophic denitrification is entirely replaced by Dissimilatory Nitrite Reduction to Ammonium (DNRA) coupled with anammox (Lam et al., 2009; Jensen et al., 2011).

**Figure 1:**
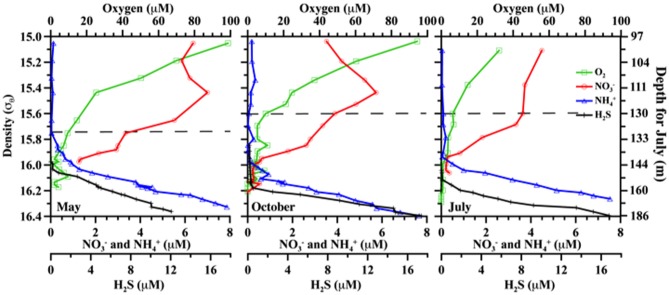
**Comparative chemical profiles for the three different cruises.** Markers indicate discrete samples. Plots are versus density as absolute depths are less consistent. For reference, July 2008 also includes absolute depth in m, but note this measure only applies to the right panel. From left to right, panels are for May 2007; October 2007; and July 2008. Horizontal dashed lines indicate the upper boundary of the suboxic zone (O_2_ = 10 μM).

The Black Sea is well suited for investigations of these processes, as it is permanently anoxic at depth and contains a well-defined suboxic zone (O_2_ < 10 μM, no detectable H_2_S; Figure 1) sandwiched between shallow oxic and deeper sulfidic waters (Murray et al., 1995). Nonetheless, there has been no consensus there as to the relative contributions of anammox and denitrification to N_2_ production (Lam et al., 2007; Fuchsman et al., 2008; Jensen et al., 2008). Some incubation experiments with Black Sea water have shown the absence of denitrification activity (Lam et al., 2007). However, the presence of both denitrifying and anammox bacteria in the Black Sea's suboxic zone have also been documented (Kirkpatrick et al., 2006; Oakley et al., 2007). In order to better understand the *in situ* activity of these organisms and its variability, we investigated transcription of metabolic genes, as a proxy for bacterial activity, from three different cruises in different seasons.

## Materials and methods

Field sampling was conducted in the northeastern Black Sea at 44° 25′ N, 37° 30′ E, onboard the *R/V Akvanavt* and *R/V Ashamba* (mean water depth >1000 m), for three time points: May 2007, October 2007, and July 2008. Data is graphed versus potential density (σ_θ_, kg m^−3^), measured by SeaBird CTD package attached to the sampling rosette. Density was used to take into account spatial and temporal effects, which may alter the absolute depth of a feature in meters. For example, the oxycline may appear tens of meters shallower or deeper at different times and/or stations, but can be consistently found at the same density range (Murray et al., 1995). Samples were taken for the suboxic zone, roughly 15.6≤σ_θ_≤16.1 (Figure 1; includes July 2008 depth comparison).

### Nutrients, dissolved gases, and productivity

Dissolved oxygen and hydrogen sulfide were measured onshore the night after daytime sampling, using standard techniques (Grasshoff et al., 1983). Nutrients were also measured (NO_3_^−^, NO_2_^−^, NH_4_^+^), with conventional chemical techniques and utilizing an autoanalyzer (Fuchsman et al., 2008). Dissolved gases were collected in evacuated 250 mL glass cylinders with HgCl_2_ pre-added, transported to the University of Washington, and measured by a Finnegan Delta XL isotope ratio mass spectrometer as per Fuchsman et al. (2008) and Emerson et al. (1999). A known amount of ^36^Ar was added to the samples (Nicholson et al., 2010). δ^18^O-O_2_ values were corrected for addition of ^36^Ar. δ^18^O-H_2_O was measured using an automated Micromass 903 mass spectrometer with CO_2_ equilibration system at the Quaternary Research Center, University of Washington. Net community productivity (NCP) calculations based on O_2_/Ar were calculated as per equation (2) of Stanley et al. (2010);

$$\text{NCP}=\Delta ({\text{O}}_{2}/\text{Ar}){\left[{\text{O}}_{2}\right]}_{\text{eq}}\;\rho \;\text{k}$$

where [O_2_]_eq_ is O_2_ equilibrium concentration (calculated from CTD temperature and salinity data), ρ is the measured density from the CTD, and k is the gas transfer velocity. Gas exchange parameters were estimated via the parameterizations of Nightingale et al. (2000), using 14 day averaged QuikSCAT wind products. This calculation assumes a well-mixed layer, negligible impact from advection or cross-diapycnal mixing (upwelling), and a steady state system.

### RNA sampling and analysis

Different versions of nitrite reductase (*nirS*) mRNA transcripts corresponding to denitrification and anammox organisms [specifically, “*Candidatus* Scalindua”-type (Lam et al., 2009)] were extracted, reverse transcribed, amplified and sequenced. Samples for RNA analysis were taken every 0.1 density level from σ_θ_ = 15.5 to 16.1, filtered directly from Niskin bottles onto 0.2 μm Millipore Sterivex™ filters, and fixed with RNALater® within 30 min of the start of filtration. October 2007 was unusual in that opportunistic RNA sampling extended deeper, to σ_θ_ = 16.3. Filters were incubated for ~1 h, frozen, shipped on dry ice to the University of Washington, and ultimately stored at −80°C. RNA extraction was conducted similar to Poretsky et al. (2005), and reverse-transcribed with random primers using the Fermentas Maxima® kit. Amplification of Scalindua-type *nirS* was performed with primers Scnir372F and Scnir845R (Lam et al., 2009). For conventional *nirS* several primer sets were tried (Braker et al., 1998; Michotey et al., 2000; Throbäck et al., 2004; see discussion). The primary results presented here are the nirS1F/6R primers of Braker et al. (1998) amplified as per Santoro et al. (2006) but using Fermentas DreamTaq™ 2x Mastermix with BSA added to 1x concentration, because it returned *nirS* type sequences for the largest number of samples. Attempts were also made to amplify different version of nitrite reductase (*nirK*) for all samples, but because amplification was at best erratic, and failed outright in the majority of cases, those results are not considered here. Scalindua-type products, uniformly single-banded, were cleaned with a Qiagen PCR Clean-up Kit, while other *nirS* products were commonly multi-banded and therefore gel-purified (Fermentas GeneJet™ Gel Purification Kit). Cloning was conducted with the StrataClone PCR Cloning Kit, and sequenced at the High-Throughput Genomics Unit (www.htseq.org). Sequences were hand-checked and amino acid translation was performed with Transeq (European Bioinformatics Institute), related protein sequences added for reference from GenBank (National Center for Biotechnology Information), and alignments performed with ClustalX. Bootstrapped data sets (Phylip's seqboot, 100 replicates) were analyzed for maximum likelihood phylogeny with Phylip (Felsenstein, 1989), and consensus tree branch lengths subsequently determined via protein maximum likelihood (JTT algorithm; Jones et al., 1992). Final tree visualization was accomplished using the program FigTree (http://tree.bio.ed.ac.uk/). Genbank accession numbers are JX102246—JX102470.

### TRFLP

DNA was extracted using a combination of standard freeze-thaw and enzymatic lysis methods, followed by phenol-chloroform extraction and spin-column purification (Qiagen) (Fuchsman et al., 2012). Amplification was obtained using Planctomycetes primers 58F (labeled) and 926R (Wang et al., 2002). PCR products were amplified for 30 cycles at 60°C (Fuchsman et al., 2012). Column purified PCR products were digested separately overnight with restriction enzymes *HaeIII*, *Hpy1881*, *MspI*, and immediately precipitated with ethanol. Fragment analysis was performed on a MegaBACE 1000 apparatus (Molecular Dynamics) at the University of Washington Marine Molecular Biotechnology Laboratory. Electrophoretic profiles were visualized with Dax software (Van Mierlo Software Consultancy, The Netherlands). TRFLP profiles were normalized downward by total peak height. Scalindua was identified as peak 236 using *HaeIII*, peak 530 using *HpyI881*, and peak 260 using *MspI*.

## Results

**Figure 2:**
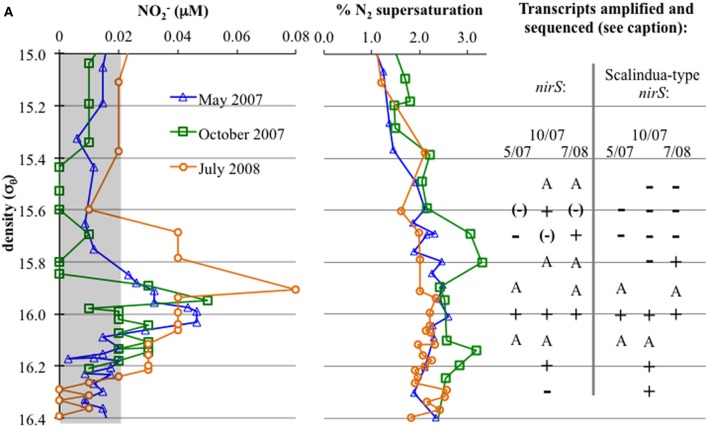
**Left:** NO_2_^−^ concentrations, with a gray box indicating values at or below detection limit. **Right:** First, N_2_ supersaturation (from N_2_/Ar ratio compared to calculated values for saturation); N_2_ is produced *in situ* in the lower suboxic zone. To the right, detection of gene transcripts: “A” indicates amplification without sequencing confirmation, while “+” indicates amplification and sequencing confirmation. “−” indicates no amplification, while “(−)” indicates bands were apparent but sequencing resulted in non-specific sequences (generally, 23S ribosomal sequences).

Nutrients (O_2_, H_2_S, NO_3_^−^, NH_4_^+^) are given in Figure 1. Oxygen penetration was deepest in May 2007 and shallowest in July 2008, affecting the apparent range of the suboxic zone (defined as O_2_ < 10 μM, H_2_S undetectable; appx. 15.7≤ σ_θ_≤16.1). Nitrate concentrations were highest at the top of the suboxic zone (max between 4.5 and 7 μM), but nitrate was detectable until at least σ_θ_ = 16.0. Nitrite was below 0.1 μM for all seasons, with a small peak around in the suboxic zone and migrating slightly in a similar fashion to oxygen (Figure 2). Ammonium concentrations were highest in the sulfidic zone, but became negligible around σ_θ_ =16.0 in October 2007 and July 2008 and at 15.8 in May 2007 (Figure 1). N_2_ supersaturation generally increased with depth and reached a broad maximum in the suboxic zone (Figure 2). N_2_ supersaturation was greatest in October 2007 with a maximum at σ_θ_ = 15.7–15.8.

**Figure 3:**
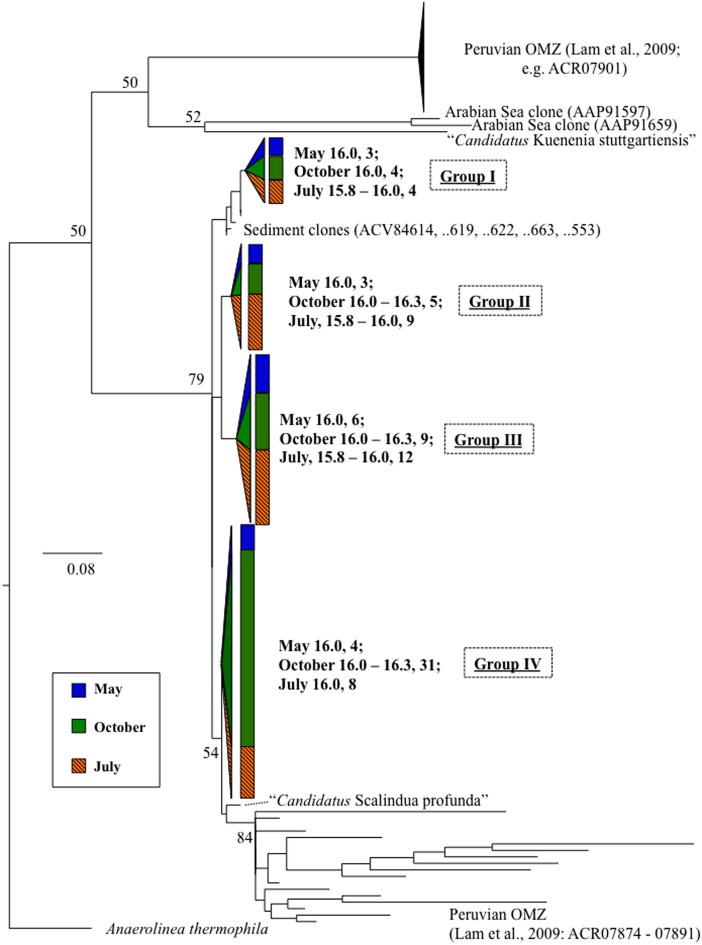
**Scalindua-type *nirS* expression, in bold, as detected with primers Scnir372F and Scnir845R (Lam et al., 2009).** Numbers indicate density, followed by number of clones. References include cultured strains (in italics), and environmental clones. “*Candidatus* Scalindua” is from van de Vossenberg et al. (2012). Tree was constructed from amino acid sequences (~160 aa), bootstrapped (100 replicates; nodes present >50% noted) and analyzed for maximum likelihood phylogeny with Phylip (Felsenstein, 1989). Scale bar indicates branch length as calculated by protein maximum likelihood (Jones-Taylor-Thornton algorithm; Jones et al., 1992). Wedge and bar sizes are proportional to clone library composition.

**Figure 4:**
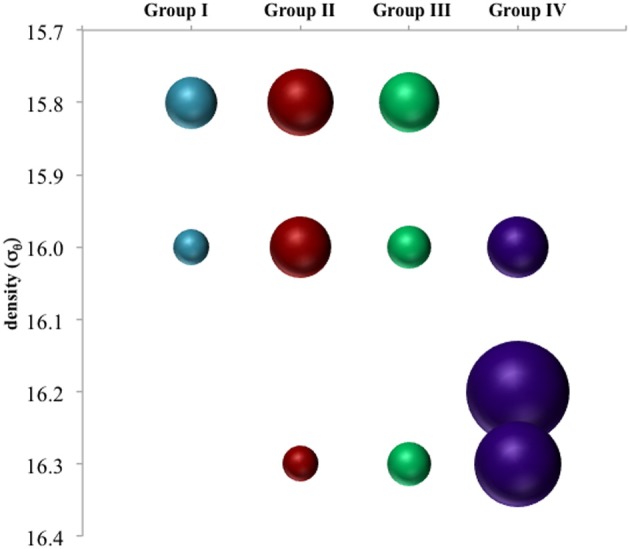
**Depth breakdown of groups noted in Figure 3.** Area of bubbles are proportional to percentage of clone library sequences for a given depth found in a given group.

**Figure 5:**
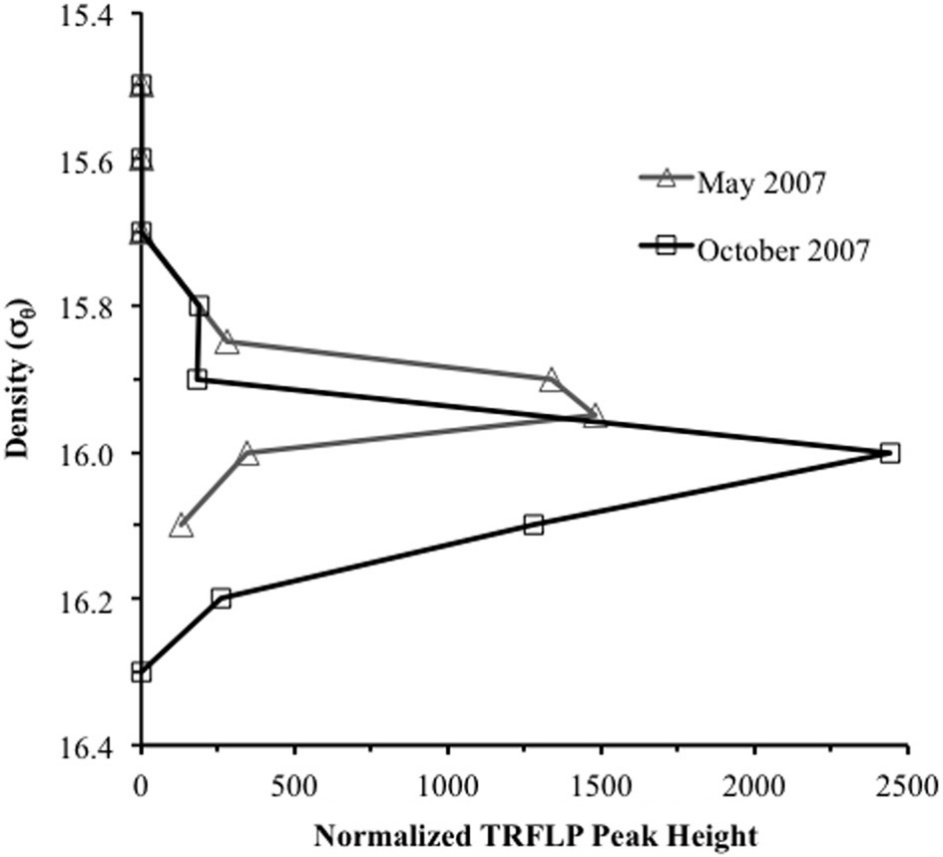
**Terminal restriction fragment length polymorphism (TRFLP) profile of “*Candidatus* Scalindua” 16S ribosomal DNA for May and October 2007.** Products were amplified using Planctomycetes specific primers 58F and 926R, and peak identification obtained with enzymes *HaeIII*, *Hpy1881*, *MspI*. Profiles were normalized downward by total peak height.

Anammox and denitrifying mRNA transcripts were detected in all three sample sets (Figure 2). Note however, that deep RNA samples (σ_θ_ = 16.2 and 16.3), extending into the sulfidic zone, were only available for October 2007. Considering first anammox, Scalindua-type *nirS* appeared localized to the lower suboxic zone (σ_θ_ = 15.8), a distribution almost invariant for the time period sampled and coincident with a stable maxima in dissolved N_2_ (Figure 2). Four sequence groups were detected (Figure 3). Groups II and III were detected across a broad depth range (Figure 4), while group I was relatively shallow (15.8 ≤ σ_θ_ ≤ 16.0) and group IV was relatively deep (16.0 ≤ σ_θ_ ≤ 16.3). TRFLP of Scalindua 16S rDNA from May and October 2007 ranged from σ_θ_ = 15.8 to 16.2 with a maximum at σ_θ_ = 15.95 in May and σ_θ_ = 16.0 in October (Figure 5).

**Figure 6:**
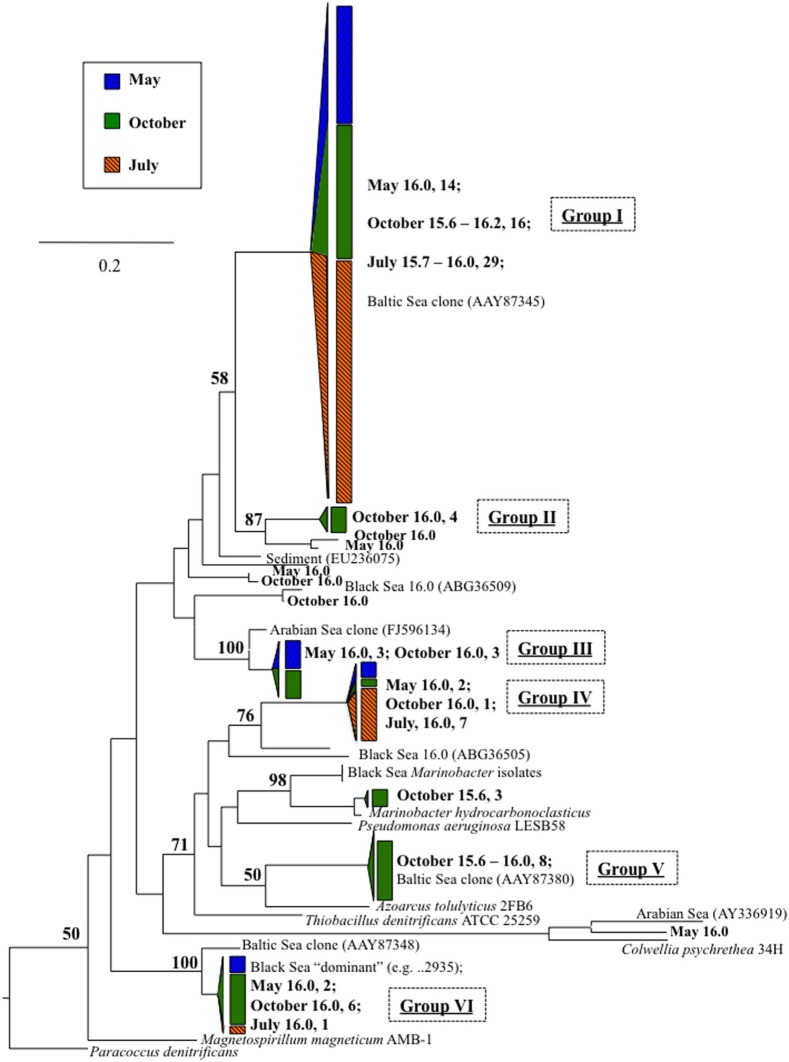
***nirS* expression as detected with degenerate primers nirS1F/6R (Braker et al., 1998).** Numbers indicate density, followed by number of clones. References include cultured strains (in italics), Baltic Sea environmental clones (Hannig et al., 2006), and DNA-based sequences previously obtained from the Black Sea (Oakley et al., 2007). Tree was constructed from amino acid sequences (~290 aa) as per Figure 3.

**Figure 7:**
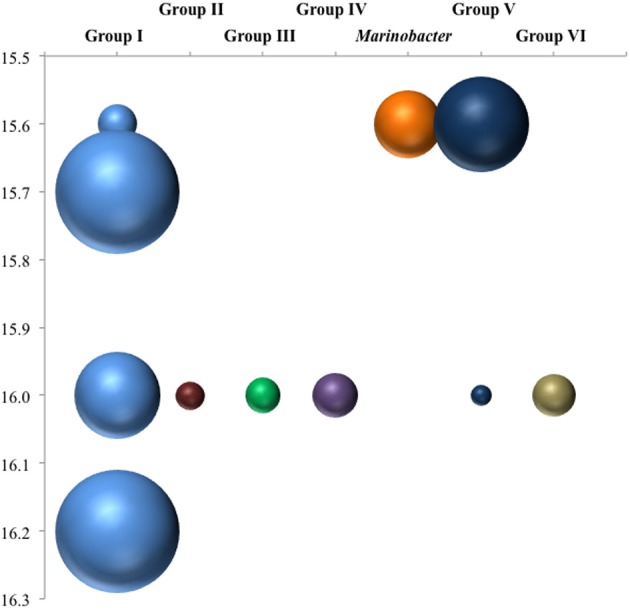
**Depth breakdown of groups noted in Figure 6.** Areas of bubbles are proportional to percentage of clone library sequences for a given depth found in a given group.

Conventional *nirS* expression was also continually present in the lower suboxic zone and continued into the upper sulfidic zone (Figure 2). Six major groups, as well as a variety of singletons, were found using the nirS1F/6R primer set and are labeled in Figure 6 for ease of discussion. Groups I, IV, and VI were expressed in a consistent pattern similar to anammox (Figure 7). Other groups and singletons were more dynamic, with varying depth ranges and/or seasons in which they were detected. Unlike anammox, however, some expression was detected in the upper suboxic zone (σ_θ_ = 15.6, 15.7; Figures 2, 7). Groups I, V, and VI were related to Baltic Sea samples (Hannig et al., 2006). Groups II and IV contained no closely related database sequences, while Group III had an Arabian Sea analog (Jayakumar et al., 2009b). Finally, *Marinobacter hydrocarbonoclasticus* was the sole described species closely related to sequences from these clone libraries.

**Figure 8:**
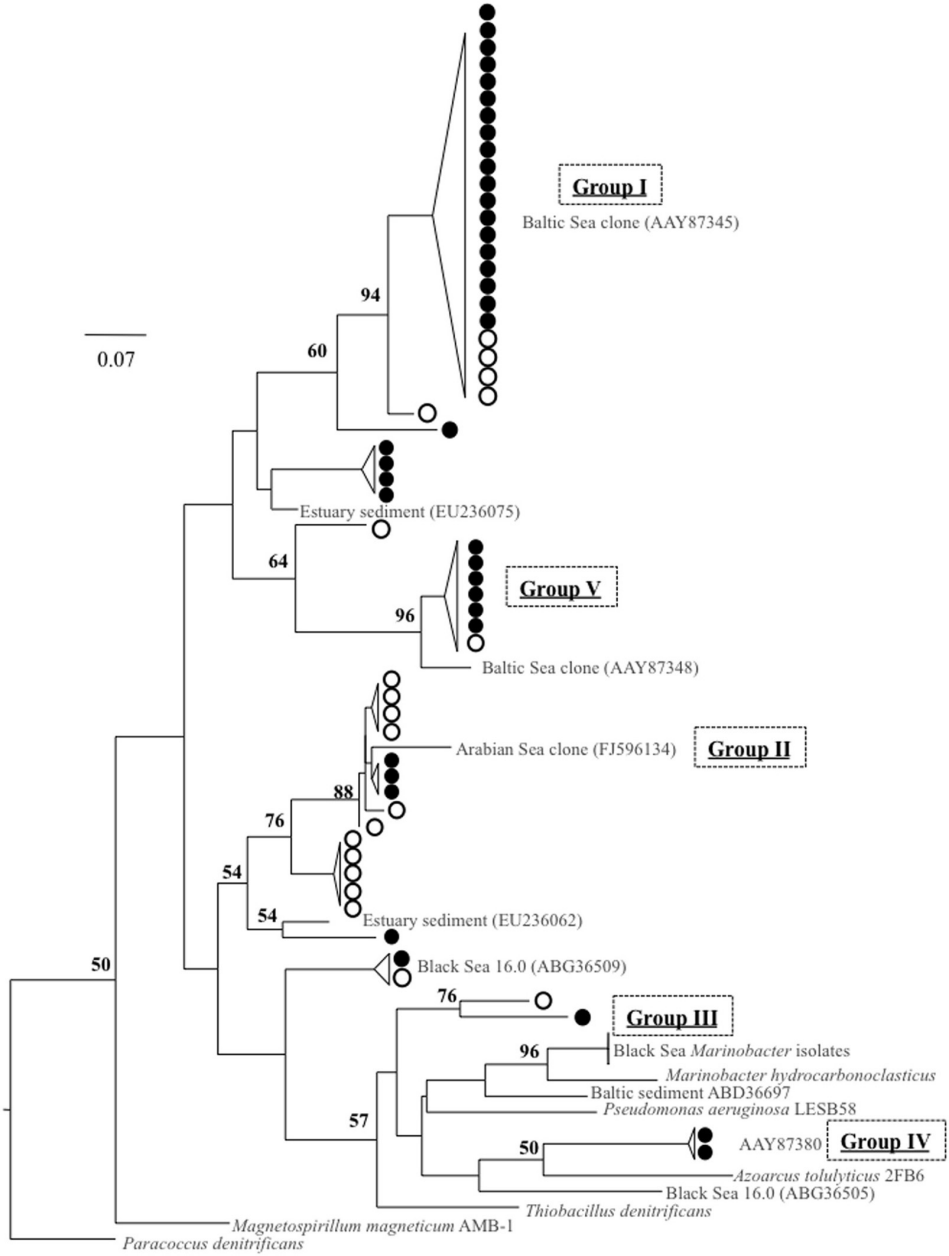
**Clone library comparison tree for two different primer pairs: nirS1F/6R, indicated by filled circles, and cd3aF/R3cd, in empty circles.** All are from October 2007, σ_θ_ = 16.0 or 16.2. References, in gray, include cultured strains (in italics), Baltic Sea environmental clones (Hannig et al., 2006), and DNA-based sequences previously obtained from the Black Sea (Oakley et al., 2007). Tree was constructed from amino acid sequences (~140 aa, necessarily shorter than other alignments) as per Figure 3.

Amplification with a second primer set for conventional *nirS* expression, cd3aF/R3cd (Throbäck et al., 2004), was only detected in the lower suboxic zone (σ_θ_ ≥ 15.9). Groups I, III, and VI from the nirS1F/6R primer set results were also found with cd3aF/R3cd (Figure 8).

**Figure 9:**
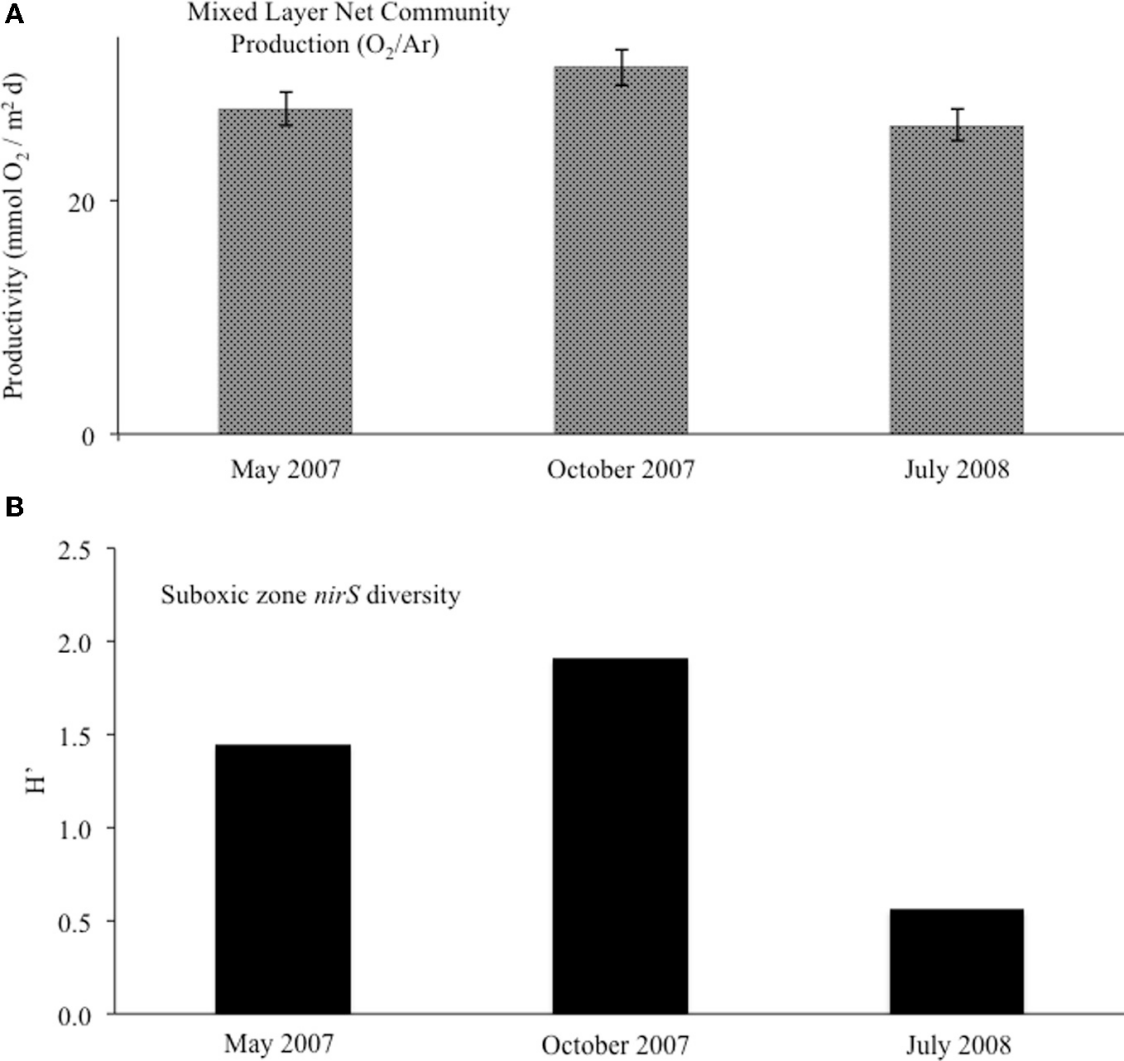
**(A)** For the surface mixed layer, net community productivity calculated with O_2_/Ar measurements. Error bars for NCP were calculated from averages of mixed layer O_2_/Ar (*n* = 4). **(B)** Suboxic zone (15.6 ≤ σ_θ_ ≤ 16.0) clone library diversity, as calculated with the Shannon-Wiener index.

**Figure 10:**
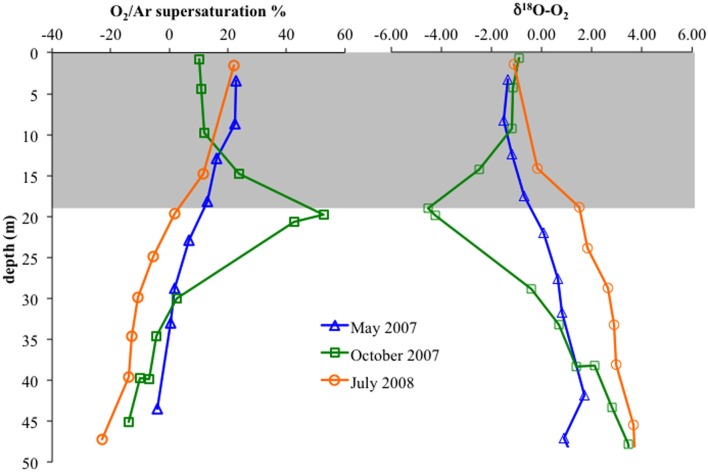
**Left:** O_2_/Ar supersaturation versus depth in the upper 50 m for May 2007, October 2007, and July 2008. **Right:** δ^18^O-O_2_ versus depth. Gray box indicates average mixed layer depths.

We used dissolved gases to estimate NCP in the surface mixed layer. NCP, effectively oxygen production in excess of consumption, was calculated from O_2_/Ar (Stanley et al., 2010; Figure 9A). NCP for the three cruises showed small but significant difference, with May 2007 and July 2008 having lower values (28 and 26 mmol O_2_ m^−2^ d^−1^) and October 2007 showing slightly elevated NCP (31 mmol O_2_ m^−2^ d^−1^). δ^18^O-O_2_ was also measured, which can show negative deviations if photosynthetic production is great enough to drive the below equilibrium levels (Quay et al., 1993). Unlike May 2007 and July 2008, October 2007 exhibited a large negative deviation in δ^18^O-O_2_ immediately below the mixed layer, concordant with a 52% supersaturation of O_2_ relative to Ar (Ar used to normalize for physical processes; Figure 10).

## Discussion

### Anammox-type expression

Anammox-type *nirS* was first used as a process-specific sequence type in the Peruvian OMZ (Lam et al., 2009). All of the sequences amplified in this Black Sea study were monophyletic with Peruvian OMZ sequences, in turn closely allied to the environmental anammox clade, “*Candidatus* Scalindua” (Lam et al., 2009; van de Vossenberg et al., 2012) (Figure 3). For comparison, anammox-type 16S rDNA was also analyzed (Figure 5). The observed distribution of this anammox-type *nirS* was consistent with this ribosomal DNA based distribution, as well as previous data sets (Kuypers et al., 2003; Kirkpatrick et al., 2006; Fuchsman et al., 2012). The presence of anammox bacteria in the lower suboxic zone may be due to the flux of ammonium from the sulfide zone that affects these depths. Although amplification of sequence types not involved in the anammox process cannot be ruled out, both the sequence data and depth distribution similarity lend credence to the applicability of these products as process specific markers.

Phylogenetically, expressed anammox-type *nirS* sequences fell into four highly similar groups (Figure 3). Distance between these Black Sea groups was less than between sequences from the Peruvian OMZ. It may be that a relatively small subset of marine anammox bacteria are adapted to the brackish waters of the Black Sea (suboxic zone salinity ~20–21) as compared to the Peruvian OMZ; this is consistent with previous 16S-based approaches (Schmid et al., 2007).

There appeared to be little difference between time points for the anammox-type *nirS* data, suggesting that these organisms were consistently transcribing this *nirS*-type gene. The single exception was July 2008, the only time that amplification could be detected as shallow as σ_θ_ = 15.8, though only a few sequences could be retrieved (Figure 2). This was also the time of shallowest oxygen penetration (Figure 1). Between groups, the only variation appeared to be associated with differences in depth. Group I expression was only detected for 15.8 ≥ σ_θ_ ≥ 16.0 and Group IV was found for 16.0 ≥ σ_θ_ ≥ 16.3; Groups II and III were found variously across the whole range (Figure 4). Interestingly, for the one sample set which extended into the sulfidic zone (October 2007) anammox activity could still be detected as deep as σ_θ_ = 16.3, where H_2_S ≥ 10 μM. Evidence for the presence of anammox bacteria coincident with low levels of sulfide has been previously found in the Black Sea (Wakeham et al., 2007; Fuchsman et al., 2012). This suggests some sulfide tolerance for Groups II, III, and IV. Theoretically, these groups could be actively involved in S-linked processes (Kalyuzhnyi et al., 2006), provided that a flux of NO_3_^−^ or NO_2_^−^ penetrates down to these density surfaces. While this is speculative, it is known that NO_2_^−^ levels for σ_θ_ ≥ 16.2 were below the detection limit for October 2007, when deep activity was sampled (Figure 2). Other explanations for anammox activity require a supply of nitrite such as horizontal advection (Stunzhas and Yakushev, 2006) or *in situ* production with sparse oxidants such as trace levels of MnOx; a fast sinking mechanism to import active cells and their mRNA; or perhaps the utilization of abundant sulfate as the ultimate electron acceptor (Liu et al., 2008). While the specific adaptations of the four different anammox types are speculative, it is nonetheless apparent that there are some micro-heterogenities in this population.

### Denitrification and relation to environmental factors

Expression patterns of denitrification appeared more complex than anammox. October 2007 stands out in this data set due several factors, including deeper samples that were not available for the other two timepoints. Even excluding these samples, however, several “intermittent” groups were detected in October 2007 that were not found other times. Denitrifiers in the upper suboxic zone (σ_θ_ = 15.6) were not only active, unlike other seasons, but included *Marinobacter* and Group V phylotypes not found at other times (Figures 6, 7). Group V contains sequences up to 100% similar to a sequence known from the Baltic Sea's upper, oxic waters (AAY87380; Hannig et al., 2006), suggesting a tolerance for relatively oxidizing conditions. The *Marinobacter*-type sequences were distinct from strains previously isolated from the Black Sea (bootstrap support 98%) (Oakley et al., 2007). July 2008 also had transcripts at a relatively shallow density surface (σ_θ_ = 15.7), but unlike the varied phylogeny of October 2007, the shallower July expression was only an extension of consistent, deeper Group I activity. This was perhaps in response to the shoaling of the oxycline (Figure 1).

Interestingly, October 2007 surface waters exhibited relatively high biological productivity (Figure 9A). October 2007 also had a large dissolved oxygen minimum below the mixed layer (Figure 10), suggestive of enhanced productivity, though assessing a rate to this deeper production is problematic. Although the linkage is indirect, if either mixed layer or deeper productivity resulted in sinking organic matter, this could have impacted heterotrophic denitrification. October 2007 was also unusual in the detection of elevated N_2_ supersaturation levels, indicative of biological N_2_ production, in both the upper suboxic zone (15.6 ≤ σ_θ_ ≤ 15.8) and the upper sulfidic layer (16.1 ≤ σ_θ_ ≤ 16.25) (Figure 2). Part of this N_2_ build-up was potentially a result of these intermittently detected denitrifiers, particularly in the upper suboxic zone (σ_θ_ < 15.8) where anammox type *nirS* expression was not found for our three time points. The deeper N_2_ peak (σ_θ_ = 16.1–16.2) was found at the transition from suboxic to sulfidic zones, and nutrient profiles (Figure 1) are suggestive of deeper H_2_S consumption in October; it is possible that autotrophic denitrification could also have been enhanced at this time.

Looking at the diversity of denitrifiers, the Shannon-Wiener index for the suboxic zone also peaked in October (Figure 9B; H′_May_ = 1.4, H′_Oct_ = 1.9, H′_Jul_ = 0.6). This is excluding the deep samples unique to the October cruise. In October, when the N_2_ build-up had both a shallow and a deep peak (Figure 2), σ_θ_ = 15.6 in the upper suboxic zone had some *nirS* expression not seen other times (Figure 7), in addition to the consistent deep expression. This multiplicity of apparent niches was one of the drivers of diversity in October. If in fact greater productivity led to C export which stimulated denitrification at depth, this would be consistent with other studies that have linked organic C input to changes in denitrification activity (Engström et al., 2005; Fuchsman et al., 2008; Ward et al., 2008, 2009).

Many denitrifier groups, on the other hand, seem more similar to the detected anammox expression in that their expression was detected regularly between seasons and in the lower suboxic zone. These “consistent” denitrifiers include an unknown type (Group IV), a Group that was previously known from Black Sea clone libraries as the “dominant” type (Oakley et al., 2007)—here, Group VI—and a third type (Group I) identical to a different Baltic Sea clone, this one from suboxic waters (AAY87345; Hannig et al., 2006). Transcripts of this Baltic Sea type were found into the sulfidic layer. Whether this represents autotrophic or heterotrophic denitrification is unknown. While there were some sequences shared with Baltic Sea studies, it is notable that the consistent expression pattern typical of Black Sea groups I, IV, and VI discussed here are different from the temporal changes documented in the Baltic Sea (Hannig et al., 2007). Denitrification was indicated in the Baltic mainly in instances where the suboxic zone was compressed or absent and nitrate and sulfide overlapped, with the establishment of a stable suboxic zone favoring anammox (Hannig et al., 2007). Measurable levels of nitrate and sulfide do not commonly overlap in the Black Sea (Murray et al., 1995), though as noted above and in Figure 1 some variability was observed in this study. Several lines of published evidence point toward autotrophic metabolism by ε- and γ-proteobacteria in the sulfidic zone, including the autotrophic denitrifier *Sulfurimonas* (Grote et al., 2008; Glaubitz et al., 2010). In the case of *Sulfurimonas*, primer mismatch could have prevented detection of mRNA transcripts by our methods; see primer bias considerations, below.

### Concurrent denitrification and anammox activity

Both denitrification and anammox type *nirS* expression were consistently detected at the same density surfaces in the lower suboxic zone. This occurs despite the fact that they have different metabolic requirements, and in spite of the restricted, relatively stable environment formed by the redox gradient; this seems to indicate that conditions are conducive for both processes to persist simultaneously. As both require nitrite, a competitive playoff between the two processes is often assumed (e.g., Hannig et al., 2007; Bulow et al., 2010; Lam et al., 2011). Our data set suggests that this may not be the case, with the ongoing activity of both processes more similar to the balanced activity seen in Skagerrak sediments (Thamdrup and Dalsgaard, 2002) or the waters of Golfo Dulce (Dalsgaard et al., 2003).

### Primer bias considerations

Regarding primer sets, it should be noted that the *nirS* primer set used here (nirS1F/nirS6R), while degenerate, is not considered universal (Throbäck et al., 2004). Nitrite reductase genes, here focusing on *nirS*, are fairly diverse and exhibit significant sequence divergence, making whole-community analysis difficult and subject to bias. A fairly comprehensive analysis of different primer sets was conducted in 2004 for cultured organisms and soil samples (Throbäck et al., 2004). Studies of ocean OMZs have commonly relied on nirS1F/6R (Braker et al., 1998) or cd3aF/R3cd (Michotey et al., 2000; Throbäck et al., 2004). In order to assess possible primer pair bias in our data sets, products of both primer sets were analyzed. It must be noted that while we could compare between primer sets and samples, it is difficult to infer what other potential sequences were missed entirely. There could have been a large number of organisms contributing to the mRNA pool that remained undetected due to primer mismatches, including *Sulfurimonas*, which has previously been detected in the Black Sea (Grote et al., 2008). Compared to the Braker primers (Braker et al., 1998), we found *Sulfurimonas denitrificans DSM1* to have 6 mismatches and one deletion over the 18 bp of nirS1F and 10 mismatches over the 16 bp of nirS6R. Regardless of what was missed, given the data at hand it was possible to compare data produced by these two primer sets in an effort to understand what relative biases may have been present.

While nirS1F/6R amplified sequences throughout the suboxic zone, albeit intermittently for the upper suboxic zone (Figure 2), cd3aF/R3cd amplification was only successful in the lower suboxic zone (σ_θ_ ≥ 15.9). This suggests that the upper suboxic zone community may be “missed” not only because of changes in activity but also due to poor amplification if analyzed with a different primer set. This was further indicated by analyzing the entire nirS1F/6R data set (all depths) for mismatches to the cd3aF/R3cd priming site, both of which are internal to the amplicon produced by nirS1F/6R. 73% of nirS1F/6R clones mismatched the cd3aF primer, and 99% mismatched R3cd. The same analysis for nirS1F or 6R binding sites is not possible, as both sites are outside of the amplicons produced by cd3aF/R3cd.

In order to check for systematic discrimination of specific phyla by the different primer sets, sequences of both for October samples σ_θ_ = 16.0 and 16.2 were obtained (Figure 8). It is important to note that ribosomal contamination was intermittently found in both data sets. This contamination, typically found in the absence of *nirS* template, was detected when sequences contained multiple stop codons and could not be aligned; it was confirmed with BLAST searches to the Genbank database. For nirS1F/6R this only happened in the upper suboxic zone. This was true even when bands of the proper size were excised and purified from the agarose gel and even when, additionally, mRNA purification of the RNA extract (MICROBExpress^©^, Ambion) was conducted before reverse transcription. This suggests that qPCR methods cannot be easily applied to these primer sets. Considering phylogeny, groups I, II, and V were present in both data sets, suggesting fairly good overlap between primer products (Figure 8). While it is not easy to make conclusions about groups that were not present, due to sampling depth, overall diversity of the two different data sets is similar (Shannon-Wiener index of 1.6 for 1F/6R, 1.8 for cd3aF/R3cd). In summary, while no clade-specific bias appeared when comparing sequences from successfully amplified PCR products of both primer sets for the same depths, for other depths nirS1F/6R was the only primer set that produced any *nirS*-type sequences at all. This suggests that, for some marine environments such as OMZs, cd3aF/R3cd may undersample diversity.

## Conclusions

Our results, based on analysis of dissimilatory nitrite reductase (*nirS*) expression over three sampling seasons, revealed that both denitrification and anammox were consistently found in the lower suboxic zone, for three sample sets spanning15 months. Consistent *nirS*-expression was localized to the lower suboxic zone, and included both anammox and conventional denitrification type genes. Intermittent *nirS* expression was detected in the upper suboxic zone (σ_θ_ ≤ 15.7) and varied between sampling times, perhaps in response to environmental variables such as oxygen and organic C input. This connection is inferred, and not proven; other studies are required to directly investigate this linkage. The fluctuating response of some denitrifiers appears not just the opportunistic response of phylotypes otherwise routinely active at other depths, but characteristic of several groups that were not found to be active at other times, within the acknowledged limitations of sampling and sequencing for this study. This underscores the problematic nature of making global N budgets based on instantaneous measurements of rate or activity, and points toward the necessity of time-integrated approaches in order to resolve conflicting estimates. Further work is needed to directly examine the interplay between surface productivity and deeper denitrification, resolve inconsistencies between methods based on *in situ* parameters versus incubations, and to understand the ongoing activity and interplay of anammox, heterotrophic denitrification, and autotrophic denitrification.

### Conflict of interest statement

The authors declare that the research was conducted in the absence of any commercial or financial relationships that could be construed as a potential conflict of interest.
